# Local Overexpression of Interleukin-11 in the Central Nervous System Limits Demyelination and Enhances Remyelination

**DOI:** 10.1155/2013/685317

**Published:** 2013-05-30

**Authors:** Anurag Maheshwari, Kris Janssens, Jeroen Bogie, Chris Van Den Haute, Tom Struys, Ivo Lambrichts, Veerle Baekelandt, Piet Stinissen, Jerome J. A. Hendriks, Helena Slaets, Niels Hellings

**Affiliations:** ^1^Biomedical Research Institute, School of Life Sciences, Hasselt University and Transnational University Limburg, 3590 Diepenbeek, Belgium; ^2^Laboratory for Neurobiology and Gene Therapy, Division of Molecular Medicine, Katholieke University of Leuven, 3000 Leuven, Belgium

## Abstract

Demyelination is one of the pathological hallmarks of multiple sclerosis (MS). To date, no therapy is available which directly potentiates endogenous remyelination. Interleukin-11 (IL-11), a member of the gp130 family of cytokines, is upregulated in MS lesions. Systemic IL-11 treatment was shown to ameliorate clinical symptoms in experimental autoimmune encephalomyelitis (EAE), an animal model of MS. IL-11 modulates immune cells and protects oligodendrocytes *in vitro*. In this study, the cuprizone-induced demyelination mouse model was used to elucidate effects of IL-11 on de- and remyelination, independent of the immune response. Prophylactic-lentiviral- (LV-) mediated overexpression of IL-11 in mouse brain significantly limited acute demyelination, which was accompanied with the preservation of CC1^+^ mature oligodendrocytes (OLs) and a decrease in microglial activation (Mac-2^+^). We further demonstrated that IL-11 directly reduces myelin phagocytosis *in vitro*. When IL-11 expressing LV was therapeutically applied in animals with extensive demyelination, a significant enhancement of remyelination was observed as demonstrated by Luxol Fast Blue staining and electron microscopy imaging. Our results indicate that IL-11 promotes maturation of NG2^+^ OPCs into myelinating CC1^+^ OLs and may thus explain the enhanced remyelination. Overall, we demonstrate that IL-11 is of therapeutic interest for MS and other demyelinating diseases by limiting demyelination and promoting remyelination.

## 1. Introduction

Multiple sclerosis (MS) is a chronic inflammatory disease of the central nervous system (CNS) that develops in genetically predisposed individuals where tolerance to self-antigens is broken down [1]. One of the pathological hallmarks of MS is loss of the nerve-insulating myelin sheath, which contributes to classical symptoms observed in individuals with MS including loss of sensation, motor, autonomic and neurocognitive functions [2, 3]. Infiltrated macrophages and resident microglia together with activated autoreactive lymphocytes orchestrate immune-mediated demyelination and concomitant axonal degeneration (reviewed in McFarland and Martin 2007) [4]. In addition to the release of cytotoxic cytokines or soluble toxic mediators [5, 6], microglia and infiltrated macrophages phagocytose and degrade myelin and are considered the main effector cells in MS. While there is evidence for endogenous remyelination in MS lesions, it fails in the majority of patients [7, 8].

Current treatments of MS aim to limit inflammation, thereby reducing the incidence of new lesions and clinical relapses. These approaches, so far, demonstrated little direct effects on regeneration and remyelination [9]. Endogenous remyelination is achieved through the rapid amplification of oligodendrocyte progenitor cells (OPCs) in demyelinated areas and their subsequent maturation into myelinating oligodendrocytes (OLs). However, remyelination is often ineffective in MS. While OPCs are still found in chronic MS lesions, they fail to differentiate into oligodendrocytes [10]. The reasons for this phenomenon are likely determined by intrinsic changes within the oligodendrocyte precursor cell population in combination with the specific inhibitory cues present in multiple sclerosis lesions (for detailed review, see Kotter et al. 2011 [11]). Factors that increase the survival, proliferation, migration, and differentiation of OPCs can favorably tweak the dysregulated signaling environment and thus promote remyelination. In this regard, Interleukin-11 (IL-11), a member of the gp130 cytokine family [12] with its well-established immunomodulatory and neuroprotective effects, can be considered as a potential therapeutic candidate. Like several other gp130 signaling cytokines, including leukemia inhibitory factor (LIF) and Oncostatin M (OsM), IL-11 is upregulated in MS lesions [13]. As LIF was shown to limit autoimmune-mediated demyelination in EAE [14], the upregulation of IL-11 in MS lesions could indicate its putative role in lesion regulation. IL-11 was reported to promote Th2 polarization of CD4^+^ T cells and reduce the production of toxic mediators by macrophages [15–17]. Moreover, IL-11 enhances the survival of OPCs by inducing STAT3-mediated antiapoptotic effect [13, 18]. While IL-11R*α* knockout mice show increased clinical symptoms of EAE, systemic treatment of wild-type EAE animals reduced disease, indicating that IL-11 is able to regulate autoimmune demyelination [19].

In this study, we used the cuprizone mouse model to characterize direct effects of IL-11 on de- and remyelination, independent of autoimmune modulation. Mice fed with cuprizone exhibit a reproducible and reversible demyelination accompanied by proliferation of OPCs and microglia in the midline region of corpus callosum. When returned to normal chow, spontaneous remyelination occurs as a result of OPC maturation to myelinating OLs [20, 21]. Our study reveals that CNS-targeted overexpression of IL-11 limits demyelination on one hand and in addition accelerates remyelination of demyelinated regions making it an interesting therapeutic target that directly enhances regeneration.

## 2. Materials and Methods

### 2.1. Development of Lentiviral Vectors and Analysis of Transgene Expression

Lentiviral vectors (LVs) are a powerful tool to obtain stable transgene expression in the central nervous system [22, 23]. HIV-1 derived, second generation LV vectors, encoding enhanced fluorescent protein (eGFP), and murine IL-11 were produced using triple transient transfection of 293T cells, as previously described [14]. Briefly, 293T cells were transfected with a packaging plasmid, a plasmid encoding the glycoprotein G of vesicular stomatitis virus and a transfer plasmid encoding murine IL-11 (NCBI Reference Sequence: NM_008350) or eGFP under the control of cytomegalovirus promoter. Vector particles in the supernatant were concentrated using Vivaspin 15 columns (Vivascience, Hannover, Germany), aliquoted and stored at −80°C. p24 antigen content was determined by HIV-1 p24 Core Profile ELISA (DuPont, Dreieich, Germany). To confirm transgene expression *in vitro*, HEK 293T cells were transduced with IL-11-LV stock solution corresponding to 1.045 ± 0.035 × 10^7^ pg of p24/mL, as described previously [14]. Expression of IL-11 protein was determined 72 h after transduction, by immunocytochemistry using monoclonal anti-mouse IL-11 antibody (2 *µ*g/mL) (R and D Systems; MAB418). Secretion of IL-11 in the supernatant of transduced 293T cells was measured by ELISA (R and D systems; DY418) 48 and 72 h after transduction. For the *in vivo* validation of transgene expression, mice were stereotactically injected with 4 *µ*L of IL-11-LV stock solution. After 2 weeks, their brains were isolated and processed for IL-11 immunostaining.

### 2.2. Stereotactic Administration

Mice were anesthetized with ketamine (80 mg/kg i.p.) and medetomidine (6 mg/kg i.p.) and fixed in the stereotactic apparatus (Stoelting, IL, USA). The skull was exposed through a small midline incision in the scalp. A hole was drilled in the skull at the chosen coordinates (in reference to bregma), namely, anterioposterior, −0.5 mm; lateral, −2.0 mm; and dorsoventral, −2.0 mm [24] (Figure 1 in Supplementary Material available online at http://dx.doi.org/10.1155/2013/685317). A 30 gauge needle fitted with 10 *μ*L hamilton syringe was inserted in the brain. After acclimatizing the needle for 5 min, 4 *μ*L of respective treatments (Phosphate buffer saline (PBS)/eGFP-LV/IL-11-LV) was administered at the rate of 0.25 *μ*L/min with the help of “Quintessential” stereotactic injector (Stoelting, IL, USA). The needle was kept in place for an additional 5 min before it was slowly retracted. The scalp was sutured and disinfected. Atipamezole (1 mg/kg i.p.) was administered and mice were allowed to recover at 37°C for 24 h. All procedures were performed under aseptic conditions. All animal experiments were approved by the Hasselt University ethics committee.

### 2.3. Cuprizone Treatment and Experimental Groups

**Figure 1:**
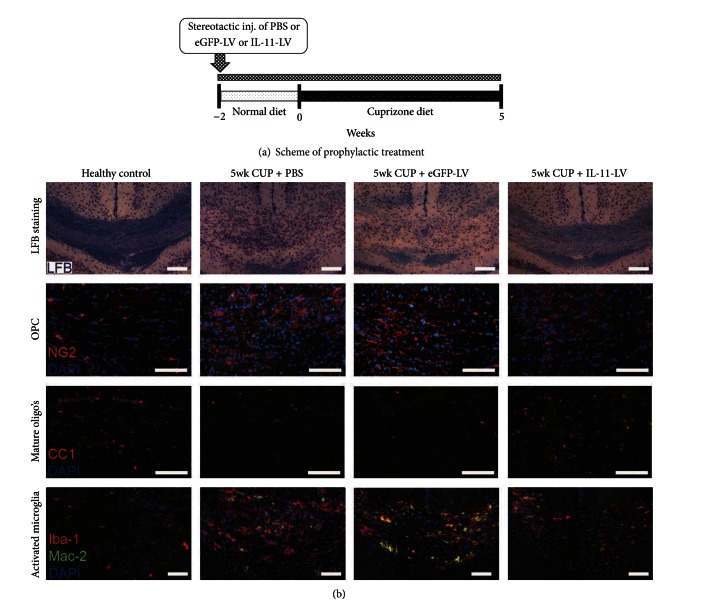
Scheme and effect of prophylactic overexpression of IL-11 on acute demyelination. (a) Murine IL-11-LV was administered stereotactically, 2 weeks prior to starting of the cuprizone (CUP) diet. PBS and eGFP-LV treatments were included as controls. After 5 weeks of cuprizone diet, animals were sacrificed and processed for immunohistochemistry. Panel (b) shows representative images of the midline corpus callosum in coronal brain sections of all groups, depicting demyelination (LFB), density of OPCs (NG2), OLs (CC1), and activated microglia (Mac-2/Iba-1). The images are chosen between the anterioposterior coordinates from −0.3 to −1.5 mm in reference to bregma. Scale bar: 200 *μ*M.

Male, adult C57BL/6J mice aged 6 to 8 weeks were purchased from Harlan. The mice were housed in a 12 h light/dark cycle at an ambient temperature and had free access to food and water. Cuprizone (Sigma Aldrich; C9012) was homogenously mixed (0.2% w/w) with powdered rodent chow and was changed daily. Two treatment regimens were chosen: (1) prophylactic overexpression of IL-11 to evaluate its effect on acute demyelination and (2) therapeutic overexpression of IL-11 (after demyelination) to evaluate its effect on remyelination. The experimental design of the two approaches is graphically illustrated in Figures 1(a) and 4(a), respectively. To induce acute demyelination, mice were fed with cuprizone diet for 5 weeks. To allow spontaneous remyelination, mice were switched to standard pelleted chow for two weeks [20, 21]. In both experiments, mice were randomly divided into three experimental groups, namely, (1) PBS treated, (2) eGFP-LV treated, and (3) IL-11-LV treated. Healthy animals were used as a control for immunohistochemistry and histology.

### 2.4. Histology and Immunohistochemistry

After completion of both study protocols, brains were excised, embedded in Tissue-Tek, and snap-frozen in liquid nitrogen. Frozen sections (10 *μ*m thick) were cut with a microtome (Leica Microsystems, Wetzelar, Germany). The myelinated area was assessed by luxol fast blue staining. To do so, acetone-fixed mouse brain coronal sections were incubated at 56°C for 16 h in luxol fast blue solution. Sections were then differentiated in 0.5% lithium carbonate solution for 45 s and counterstained with cresyl violet. Immunohistochemistry was performed using primary antibodies, namely, rat anti-IL-11 (5 *µ*g/mL; R and D systems; MAB 418), rabbit anti-NG2 (1 : 200; Millipore; AB5320), rabbit anti-Iba-1 (1 : 350; Wako chemicals; 019-19741), mouse anti-Mac-2 (1 : 250; ATCC; clone M3/38.1.2.8 HL.2), and mouse anti-CC1 (2 *µ*g/mL; Calbiochem; OP80). For all immunostainings (except CC1), sections were fixed in ice-cold acetone for 10 min and air-dried for 30 min. For CC1 immunostaining, sections were fixed in ice-cold 4% paraformaldehyde buffer (pH = 7.3) for 10 min and then kept in boiling 0.01 M citrate buffer for 30 min for antigen retrieval. Cryosections were blocked with 10% goat serum in 0.05% PBS-Tween20 (PBS-T) for 1 h and incubated with respective concentrations of primary antibody diluted in blocking buffer, for 3 h at room temperature. The sections were then incubated with goat anti-species secondary antibodies conjugated with Alexa-488, 555 or 568 (Invitrogen) for 2 h at room temperature. DAPI was used as nuclear stain. All sections from one study were treated identically side by side.

### 2.5. Transmission Electron Microscopy (TEM)

The sample preparation for TEM was performed as previously described [25] with minor modifications. Briefly, mice were transcardially perfused with ice-cold 2% glutaraldehyde in 0.05 M cacodylate buffer (pH = 7.3) under deep pentobarbitone anesthesia. A coronal brain block (1 mm thick) within the anterioposterior coordinates from −0.3 to −1.5 mm was cut in the midsagittal plane. Subsequently, tissue was postfixed in 2% osmium tetroxide for 1 h, stained with 2% uranyl acetate in 10% acetone for 20 min, dehydrated through graded concentrations of acetone, and embedded in epoxy resin (Araldite). Semithin sections (0.5 *µ*m) were stained with a solution of thionin and methylene blue (0.1% aqueous solution) for light microscopic examination to delineate the region of interest. Ultrathin sections (0.06 *µ*m) from selected tissue blocks were mounted on 0.7% formvar-coated grids, contrasted with uranyl-acetate followed by lead citrate, and examined on a Philips EM 208 transmission electron microscope (Philips, Eindhoven, The Netherlands) operated at 80 kV. 

### 2.6. Quantification

For histological quantifications, images were acquired using a Nikon eclipse 80i microscope (Nikon corporations, Japan). Adobe Photoshop CS4 was used to adjust image contrast and brightness (all images were adjusted equally within an experiment). A mosaic of images of the corpus callosum was prepared for each section. For each mouse, at least three coronal sections 150 *µ*m apart were used for analysis. These sections were obtained in the splenium and dorsal hippocampal commissure regions [26], that is, between the anterioposterior coordinates of −0.3 to −1.5 mm with respect to bregma. Quantification was performed in the region of interest (ROI) (Supplementary Figure 1) spanning the corpus callosum from the contralateral to the ipsilateral horn using ImageJ software (1.44p; http://imageJ.nih.gov/ij). The myelinated area in the corpus callosum of luxol fast blue-stained sections was quantified using a manually set threshold, equivalent to all images. Fluorescence-based morphometric cell counting was performed manually. Individual cells were counted based on the presence of nuclei. The number of myelinated fibres and G-ratio was quantified in the electron micrographs of the cross-section of the midsagittal corpus callosum (at least three mice/group). G-ratio was calculated as axon diameter/fiber diameter. Individual axons were categorized based on their individual G-ratio as spared (G-ratio < 0.74), remyelinating (0.74 ≤ G-ratio > 0.90), and demyelinated (G-ratio ≥ 0.90) axons. Images from nonserial ultrathin sections were captured at a magnification of 11kX, and five representative images were selected per mouse by a blinded observer for evaluation using ImageJ software. 

### 2.7. Myelin Phagocytosis Assay

To analyze myelin phagocytosis, the BV2 microglial cell line was plated in 24-well plates (7.5 × 10^4^ cells/well) and incubated for 2 h at 37°C and 5% CO_2_. Cells were subsequently treated for 12 h with logarithmically increasing concentrations (1, 3, 10, and 30 ng/mL) of recombinant mouse (rm) IL-11 (R and D systems; 418-ML). To the control cells, no cytokine was added. Next, the medium was changed and a myelin phagocytosis assay was performed as described previously [27]. In short, DiI-labeled myelin (50 *μ*g /well) was added to the BV2 cultures and incubated for 90 min at 37°C and 5% CO_2_. Noningested and nonbound myelin was removed by washing the plates with ice-cold PBS. Cells were detached with 5 mM EDTA in PBS for 15 min. The amount of myelin phagocytosed was determined by measuring the cellular DiI fluorescence intensity using a FACScalibur flow cytometer (BD Biosciences, Erembodegem, Belgium).

### 2.8. Statistical Methods

Data is analyzed using Graph-Pad prism (GraphPad software Inc, USA). All results are expressed as mean ± SEM. Statistical significance is assessed by one-way ANOVA followed by Bonferroni's multiple comparison test. *P* values smaller than 0.05 are considered significant.

## 3. Results

### 3.1. Prophylactic Overexpression of IL-11 Limits Cuprizone-Induced Demyelination

**Figure 2:**
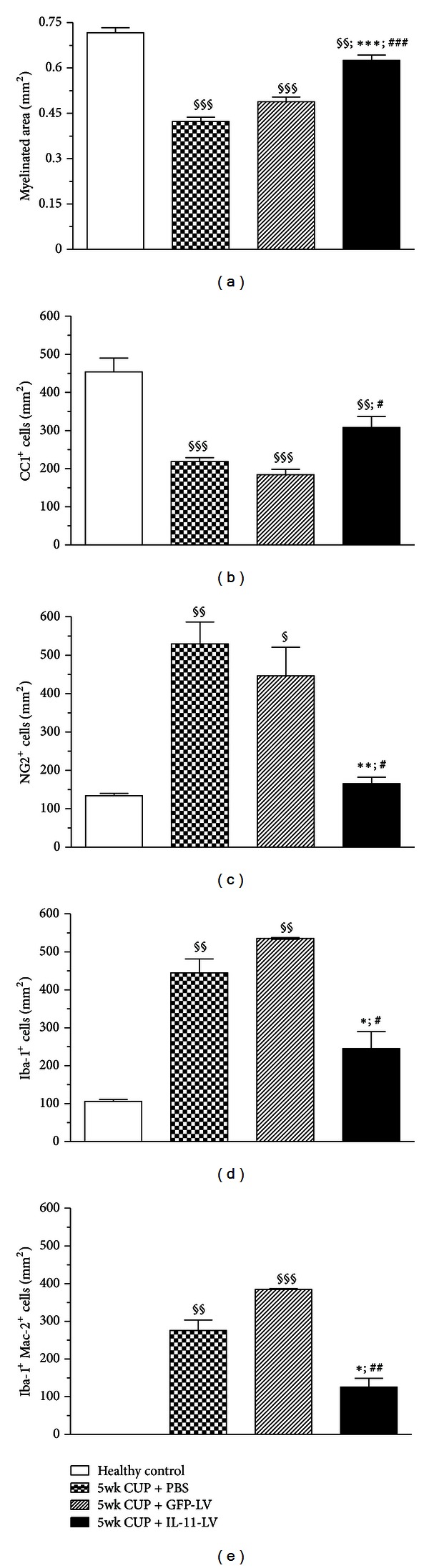
Quantification of the effect of prophylactic overexpression of IL-11 on demyelination, OPCs, OLs, and microglia. Quantitative analysis of coronal brain sections revealed that IL-11 (i) *limits demyelination*, as evident by a larger luxol fast blue-stained myelinated area (a), (ii) *prevents the degeneration of mature OLs and limits proliferation of OPC's *as demonstrated by immunostaining with CC1 (b) and NG2 (c), and (iii)* limited microgliosis (*d*) and activation of microglia *(e). Data is expressed as mean ± SEM (*n* = 5 animals per group). Statistical significance is analyzed by one-way ANOVA followed by Bonferroni's multiple comparison test. ^§^ *P* < 0.05, ^§§^ *P* < 0.01, ^§§§^ *P* < 0.001 as compared to healthy control; *P < 0.05, **P < 0.01, ***P < 0.001 compared to 5 wk CUP + PBS group; ^#^ *P* < 0.05, ^##^ *P* < 0.01, ^###^ *P* < 0.001 compared to 5 wk CUP + eGFP-LV group. Quantification was performed in the corpus callosum of at least three coronal brain sections per mouse.

First, the murine IL-11 encoding lentiviral vector (IL-11-LV) was verified for its expression *in vitro* as well as *in vivo* (Supplementary Figure 2). Consequently, the effect of prophylactic overexpression of IL-11 on acute demyelination was investigated. To allow stable overexpression, lentiviral vectors were stereotactically administered in the brain of healthy mice, 2 weeks prior to starting the cuprizone diet. PBS and eGFP-LV treatments were used as controls (Figure 1(a)). After 5 weeks of cuprizone diet, the corpus callosum appears hypercellular and edematous. Luxol fast blue staining revealed significant demyelination in the corpus callosum (Figure 1(b)). A significant (*P* < 0.001) decrease in the myelinated area was observed in the PBS and eGFP-LV treated cuprizone groups (0.4209 ± 0.01299 and 0.4884 ± 0.01548 mm^2^, resp.) as compared to healthy mice (0.7160 ± 0.01803 mm^2^). Prophylactic overexpression of IL-11 prevented cuprizone-induced demyelination by 60% as evident by a significantly larger (*P* < 0.001) myelinated area (0.6204 ± 0.02318 mm^2^) compared with the eGFP-LV treated cuprizone group (Figure 2(a)). Thus, local expression of IL-11 limits acute demyelination in the CNS.

### 3.2. Prophylactic Overexpression of IL-11 Limits Mature OLs Loss and Subsequent OPCs Proliferation

Apoptosis of mature OLs has been implicated as the primary cause of demyelination in the cuprizone model [28–30]. After 5 weeks of cuprizone diet, the density of CC1^+^ mature OLs dropped significantly (*P* < 0.001) from 453.7 ± 36.61/mm^2^ in healthy mice to 218.8 ± 9.95 and 184.3 ± 13.88/mm^2^ in PBS and eGFP-LV treated groups, respectively. Prophylactic overexpression of IL-11 significantly (*P* < 0.05) reduced cuprizone-induced loss of OLs by 46% as evidenced by a higher density (308.0 ± 28.66/mm^2^) of these cells in corpus callosum (Figures 1(b) and 2(b)). In line with the previous reports, a 4-5-fold increase of NG2^+^ OPCs was observed after 5 weeks of cuprizone diet, reflecting proliferation of OPC in response to depletion of mature OLs [21, 31]. The density of OPCs significantly (*P* < 0.05) increased from 134.1 ± 6.14/mm^2^ in healthy mice to 529.4 ± 57.31 and 446.5 ± 74.33/mm^2^ in PBS and eGFP-LV treated cuprizone groups, respectively. Prophylactic overexpression of IL-11 significantly prevented (*P* < 0.05) the proliferation of NG2^+^ OPCs as their density (165.8 ± 16.56/mm^2^) was almost equivalent to that of normal healthy values (Figures 1(b) and 2(c)). Together these data show that IL-11 prevents the cuprizone-induced OLs cell death, thereby limiting the proliferation of OPCs in the demyelinating regions. 

### 3.3. Prophylactic Overexpression of IL-11 Prevents Microglial Proliferation and Activation

Extensive microgliosis is observed in the region of cuprizone-induced demyelination. In line with earlier findings, we observed a 4-5-fold increase in the density of Iba-1^+^ microglia in the corpus callosum after 5 weeks of cuprizone diet. The density of microglia grew significantly (*P* < 0.01) from 105.9 ± 4.97/mm^2^ in the healthy control group to 444.4 ± 37.01 and 535.1 ± 2.212/mm^2^ in PBS and eGFP-LV treated groups, respectively. Prophylactic overexpression of IL-11 significantly reduced (*P* < 0.05) the proliferation of microglia by 63%, and its density decreased to 245.2 ± 44.76/mm^2^ (Figures 1(b) and 2(d)). Moreover, prophylactic overexpression of IL-11 limited the activation of microglia as shown by the density of Iba-1^+^Mac-2^+^ cells (125.7 ± 22.85/mm^2^), which was observed to be significantly lower (*P* < 0.05) as compared with the PBS (275.6 ± 27.86/mm^2^) and eGFP-LV (384.5 ± 2.38/mm^2^) treated groups. Thus, local expression of IL-11 in the CNS limits microglia activation during demyelination.

### 3.4. IL-11 Inhibits Myelin Phagocytosis by BV2 Cells **In Vitro**

**Figure 3:**
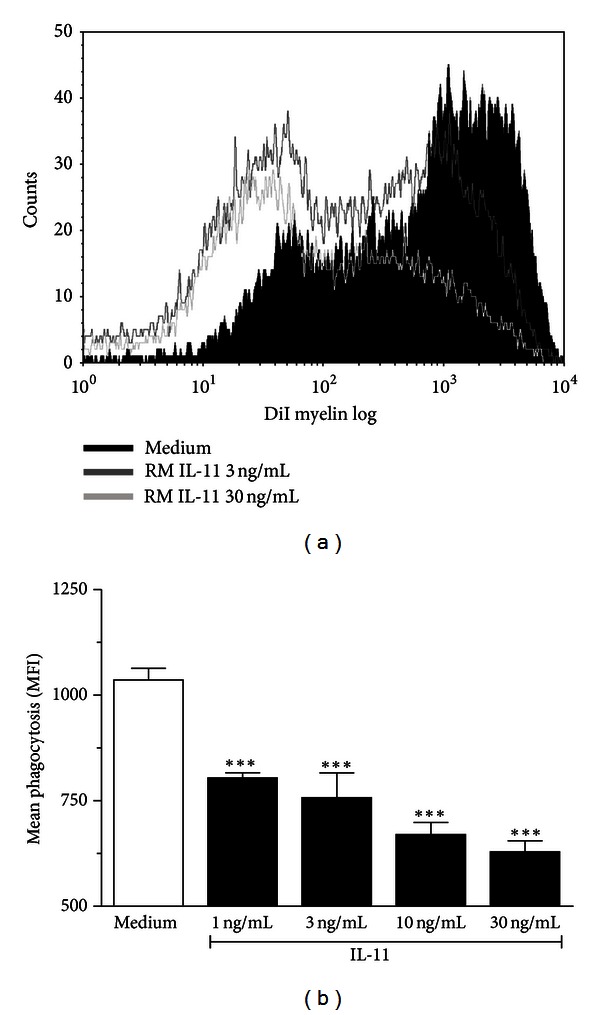
IL-11 inhibits myelin phagocytosis *in vitro.* BV2 cells were pretreated with increasing amounts of rmIL-11 for 12 h. Medium only was used as control. Subsequently cells were allowed to phagocytose DiI-labeled myelin for 90 min. (a) A representative single-parameter histogram showing IL-11-induced dose-dependent inhibition of DiI-labelled myelin phagocytosis. (b) Bar graph showing mean fluorescence intensity of DiI. The experiment was repeated three times and was performed in triplicate each time. Data is presented as mean ± SEM of triplicate determinations from three independent experiments. Statistical significance is analyzed by one-way ANOVA followed by Bonferroni's multiple comparison test. ***P < 0.001 as compared with medium only group.

To further dissect the direct effect of IL-11 on microglial activation in the context of myelin breakdown, we investigate the effect of IL-11 on myelin phagocytosis by BV2 (microglial) cells line *in vitro*. Pretreatment of BV2 cells with rmIL-11 (1, 3, 10, and 30 ng/mL) for 12 h dose dependently reduced (*P* < 0.001) subsequent phagocytosis of DiI-labeled myelin (Figure 3).

### 3.5. Therapeutic Overexpression of IL-11 Enhances Remyelination

**Figure 4:**
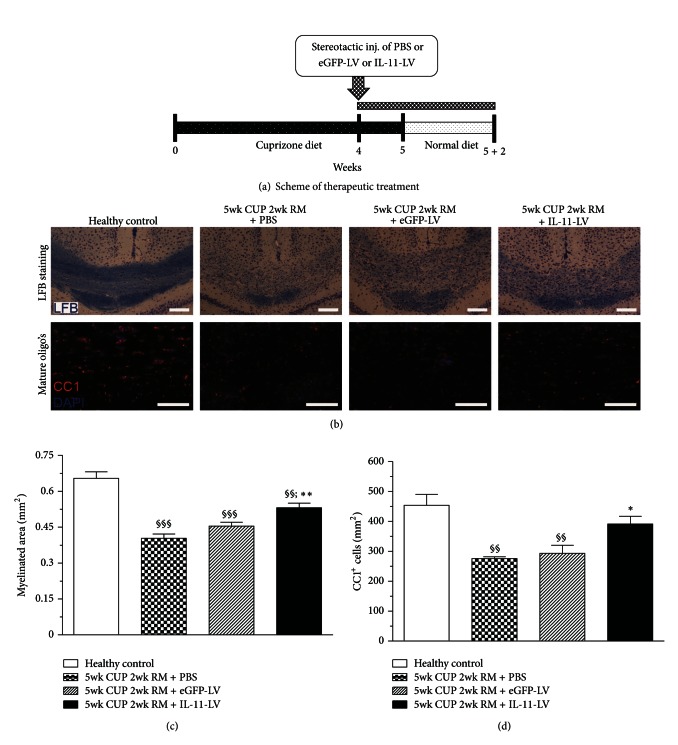
Scheme and the effect of *therapeutic overexpression of IL-11 on remyelination.* (a) Mice were fed with cuprizone diet for 4 weeks before PBS/eGFP-LV/IL-11-LV was administered stereotactically in the brain. Cuprizone diet was continued for more 1 week, before returning mice to standard chow for another 2 weeks. Panel (b) shows representative images of coronal brain sections from all groups, depicting myelinated area (LFB) and density of mature OLs (CC1). Quantitative analysis reveals that therapeutic overexpression of IL-11 (i)* enhances remyelination*, as illustrated by luxol fast blue staining depicting a larger myelinated area in the IL-11-LV treated group (c), and (ii)* facilitates the maturation of OL's *as revealed by a higher density of CC1^+^ mature oligodendrocytes in the IL-11-LV treated group (d). Data is presented as mean ± SEM (*n* = 5 animals for the cuprizone treated groups; *n* = 3 for the healthy control group). Statistical significance was analyzed by one-way ANOVA followed by Bonferroni's multiple comparison test. ^§§^ *P* < 0.01, ^§§§^ *P* < 0.001 as compared to the healthy control group; *P < 0.05, **P < 0.01, as compared to the 5 wk CUP 2 wk RM + PBS. Quantification was performed in the corpus callosum of at least three coronal brain sections per mouse, chosen between anterioposterior coordinates from 0.3 to −1.5 mm in reference to the bregma. Scale bar: 200 *µ*M.

**Figure 5:**
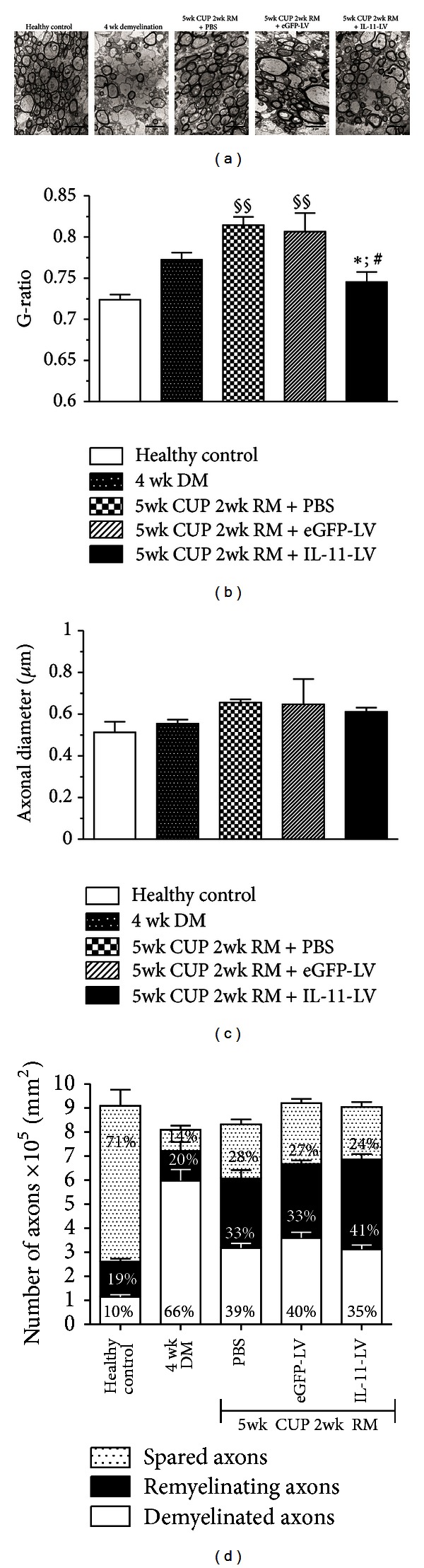
*Therapeutic overexpression of IL-11 promotes endogenous remyelination. *Representative images of electron micrographs (panel (a)) of midsagittal cross sections of the corpus callosum reveal significant demyelination after 4 weeks of cuprizone diet and remyelination after 5 weeks of cuprizone diet followed by 2 weeks of normal diet. Quantitative analysis shows that therapeutic overexpression of IL-11 (b) *enhances remyelination* as indicated by a decrease in G-ratio (c) without affecting the mean axonal diameter. (d) Quantitative and qualitative analysis reveals that 4 weeks of cuprizone treatment reduced the density of myelinated axons and again increased after the animals were allowed to remyelinate for two weeks. The values shown in the bars represent the percentage of axons of that category with respect to the total number of demyelinated and myelinated axons. Axons were categorized in different groups based on their individual G ratio: axons were defined as spared (G ratio < 0.74), remyelinating (0.74 < G ratio > 0.9), and demyelinated (G ratio > 0.90). Per mouse, quantification was performed in 5 randomly selected areas in the electron micrographs of midsagittal cross sections of the corpus callosum taken between anterioposterior coordinates from −0.3 to −1.5 mm in reference to bregma. Data is presented as mean ± SEM (*n* = 3 animals for each group). Statistical significance was analyzed by one-way ANOVA followed by Bonferroni's multiple comparison test. ^§§^ *P* < 0.01, ^§§§^ *P* < 0.001 compared to healthy control group; *P < 0.05 compared to 5 wk CUP 2 wk RM + PBS group; ^#^ *P* < 0.05 compared to 5 wk CUP 2 wk RM + eGFP-LV group; ^@^ *P* < 0.05 compared to 4 wk DM group.

We next sought to investigate the direct effect of IL-11 on endogenous remyelination. Therefore, animals were kept on a cuprizone diet for 5 weeks to induce complete demyelination. One week prior to that, IL-11-LV or respective controls were administered to allow for a sustained and maximal overexpression. Animals were then allowed to remyelinate spontaneously by switching to standard diet for 2 weeks (Figure 4(a)). Therapeutic overexpression of IL-11 showed an accelerated endogenous remyelination in the corpus callosum. As shown by luxol fast blue staining, the myelinated area in IL-11-LV treated group (0.5311 ± 0.01893 mm²) was found to be significantly (*P* < 0.01) higher as compared to the PBS treated group (0.4036 ± 0.01793 mm²). A trend of increased remyelination was observed as compared to the eGFP-LV treated group (0.4544 ± 0.01643 mm²) although not statistically significant. Therapeutic overexpression of IL-11 was able to fasten spontaneous remyelination, but not up to the extent of the healthy control group (0.6540 ± 0.02711 mm²). To further verify whether IL-11 directly affects remyelination, we analyzed ultra-thin cross sections of the corpus callosum by electron microscopy. The G-ratio (ratio of the inner axonal diameter to the total fiber diameter) is considered to be the functional and structural index of optimal axonal myelination [32]. In the IL-11-LV treated group, the G-ratio (0.7452 ± 0.012) of myelinated axons was significantly (*P* < 0.05) lower as compared to that in the PBS and eGFP-LV treated groups (0.8146 ± 0.01; 0.8066 ± 0.02), respectively. Moreover, the G-ratio of the IL-11-LV treated group was higher than that in the healthy control group (0.7238 ± 0.006) indicating that the myelin sheath is newly formed and therefore relatively thin (Figure 5(b)). The mean axonal diameter was not significantly different among the groups (Figure 5(c)). Four weeks of cuprizone diet led to a significant decrease (*P* < 0.001) in the density of myelinated axons (2.09 ± 0.53 × 10^5^/mm²) as compared to the healthy control group (7.69 ± 0.95 × 10^5^/mm²). After two weeks of endogenous remyelination, the number of myelinated axons increased in the PBS, eGFP-LV, and IL-11-LV treated groups (5.14 ± 0.53, 5.61 ± 0.21, and 5.92 ± 0.38 × 10^5^/mm^2^, resp.) without significant difference. Ninety percent of all counted axons were myelinated (spared and remyelinating) in the healthy control group. This percentage dropped significantly to 34% after 4 weeks of cuprizone diet. Following 2 weeks of spontaneous remyelination, a significant (*P* < 0.01) increase in the percentage of myelinated axons was observed in the PBS (61%), eGFP-LV (60%), and IL-11-LV treated (65%) groups (Figure 5(d)). A close observation of the micrographs showed comparatively more structured tissue architecture in the IL-11-LV treated group (Figure 5(a)). Together these data confirm the potent remyelinating capability of IL-11 *in vivo*.

### 3.6. Therapeutic Overexpression of IL-11 Promotes Maturation of OLs during Remyelination

To reveal the underlying mechanism of this therapeutic effect, we investigated the effect of IL-11 on the maturation of OLs during 2 weeks of endogenous remyelination. Therapeutic overexpression of IL-11 increased the number of mature OLs in the corpus callosum. In the IL-11-LV treated group, the density of mature OLs in corpus callosum was significantly (*P* < 0.05) increased (390.9 ± 26.32/mm^2^) as compared to the PBS and eGFP-LV treated groups (275.8 ± 5.79 and 293.3 ± 27.12/mm^2^, resp.) (Figures 4(b) and 4(d)). Moreover, the density of Iba-1^+^Mac-2^+^ microglia in the corpus callosum of IL-11-LV treated animals (181.9 ± 12.93/mm^2^) was significantly (*P* < 0.05) lower than that in the PBS and eGFP-LV treated groups (304.9 ± 20.20/mm^2^ and 278.0 ± 23.40/mm^2^, resp.) during remyelination (Supplementary Figure 3).

## 4. Discussion

The appearance of inflammatory demyelinating lesions in the white matter of MS patients is thought to result from a coordinated autoimmune attack against CNS tissue. It is common knowledge that toxic mediators such as reactive oxygen species and proinflammatory cytokines produced by infiltrating immune cells and locally activated microglia are major contributors in the formation of these myelinating lesions. It has become increasingly clear that both inflammatory cells and CNS resident cells are also able to produce factors that counteract the further spread of these lesions by promoting repair (for review see Kerschensteiner et al. 2009) [33].

Amongst others, different members of the gp130 family of cytokines are reported to be upregulated in MS lesions and likely represent an endogenous protective response mechanism to limit brain damage [13, 34]. LIF, OsM, and IL-11 are members of this family of cytokines that activate STAT3 through gp130 signaling. These cytokines exert effects on both immune cells and neural cells. Importantly, since they activate different receptors, LIF, OsM, and IL-11 can influence these parameters to different extents. During the past years, we investigated the effects of different gp130 family cytokines on distinct pathological processes in MS lesions. The experimental approach chosen is stereotactic application of lentiviral vectors to achieve a robust and stable expression and secretion within the CNS of adult mice, thereby mimicking the endogenous response in MS lesions. Moreover, this approach is able to discriminate systemic versus local effects of these neuropoietic cytokines on MS disease processes. Our study demonstrates that local overexpression of IL-11 is able to limit cuprizone-induced demyelination by reducing OLs cell death and decreasing microglial activation. Moreover, we demonstrated that IL-11 overexpression in established demyelinated brain regions enhances spontaneous remyelination.

In line with earlier observations [21, 31], a drop in the number of CC1^+^ OLs was observed in the corpus callosum after 5 weeks of cuprizone diet, paralleled with a significant increase in the number of NG2^+^ OPCs. Degeneration of OLs during cuprizone intoxication is thought to be the result of apoptosis-induced cell death [29, 31, 35]. To compensate for this OLs loss, OPCs are recruited to demyelinated areas and proliferate to replenish the OLs pool. Here we show that prophylactic overexpression of IL-11 limits mature OLs loss and prevents the consequent demyelination and proliferation of OPCs. Our *in vivo* findings are in line with previous reports demonstrating that IL-11 is promoting survival of OL in cultures through activation of STAT3 [13, 18]. Moreover, IL-11R*α* knockout mice showed enhanced OL loss and demyelination in the lysolecithin-induced focal demyelination model [18] again underscoring the importance of IL-11R signaling in promoting survival of oligodendrocyte lineage cells. In addition to IL-11, LIF has been documented to promote survival of OL by inducing antiapoptotic 14-3-3 isoforms and activating Akt-signalling [36]. Together, these reports illustrate that different members of the gp130 cytokine family may have similar effects, although different pathways could be involved.

Demyelination in the cuprizone model also partially depends on the secretion of proinflammatory cytokines by microglia which are cytotoxic to OLs and neuronal cells [37, 38]. Indeed, Pasquini et al. showed that inhibition of microglial activation by minocycline significantly reduced cuprizone-induced demyelination [37]. Moreover, *in vivo* deactivation of microglia in EAE ameliorates neurodegeneration and demyelination [39]. Our finding that IL-11 expression reduced the activation of microglia *in vivo* could therefore also explain the reduction in OL loss and consequent demyelination. In line with that, IL-11 was previously reported to inhibit tumor necrosis factor (TNF) *α*, IL-1*β*, IL-12, IL-6, and nitric oxide production by activated macrophages *in vitro* [17, 40]. Moreover, we demonstrated that IL-11 dose dependently diminished the uptake of myelin by the BV-2 microglial cell line* in vitro*. Since microglia are known to actively participate in stripping of myelin from axons, this could be a third mechanism by which IL-11 reduces demyelination. Further studies should determine in what way microglia participate in the observed IL-11-induced effects.

We further report that therapeutic overexpression of IL-11 resulted in an increased myelinated area (LFB) as compared to the control treatment groups after 2 weeks of endogenous remyelination. Furthermore, the mean G-ratio [32] of myelinated axons was significantly decreased in the IL-11 treatment group as compared to the control treatment groups. Since the mean axonal diameter was not significantly different among groups, the observed decreased G-ratio in the IL-11 treatment group reflects an increased thickness of myelin and thus enhanced remyelination. Since IL-11 increased the density of CC1^+^ mature OLs *in vivo*, the increased remyelination could result from enhanced maturation of OPCs to myelinating OLs as shown by Zhang et al. *in vitro*. Earlier *in vitro* studies already reported promyelinating effects of IL-11. Indeed, IL-11 increased the numbers and promoted the maturation of myelinating cells in CNS cocultures [13, 18].

## 5. Conclusion

Our report shows that IL-11 is of therapeutic interest for diseases with a demyelinating component. Moreover, due to its potent anti-inflammatory effects on different immune subsets (T cells, DC, and macrophages) [15, 18, 19], IL-11 seems to be an ideal tool to intervene in MS disease processes at different levels. This hypothesis is supported by the group of Gurfein et al. that reported a significant increase in EAE disease severity and neuropathology in IL-11R*α* knockout mice [19]. They further demonstrated that systemic IL-11 treatment was shown to regulate EAE disease via a combination of immunoregulation and a prosurvival effect on oligodendrocytes. In contrast, IL-11 signalling was recently found to be dispensable in spinal cord injury (SCI) [41], indicating that IL-11 may not be protective in all disease settings. 

## Supplementary Material

Supplementary Figure 1: Graphical representation of the region of interest (ROI) and the site of stereotactic injection.
Supplementary Figure 2: In vitro and in vivo validation of transgene expression (IL-11) using LV.
Supplementary Figure 3: Therapeutic overexpression of IL-11 downregulates the activation of microglia during remyelination.
