# Supplementary material for: Local Overexpression of Interleukin-11 in the Central Nervous System Limits Demyelination and Enhances Remyelination
Source: Mediators Inflamm. 2013 May 30;2013:685317. doi: 10.1155/2013/685317 (PMC3683504; doi:10.1155/2013/685317)
Supplement: Supplementary file 1 — Supplementary Figure 1: Graphical representation of the region of interest (ROI) and the site of stereotactic injection. Supplementary Figure 2: In vitro and in vivo validation of transgene expression (IL-11) using LV. Supplementary Figure 3: Therapeutic overexpression of IL-11 downregulates the activation of microglia during remyelination. [file 685317.f1.docx]

**Supplementary figures with legends**

***
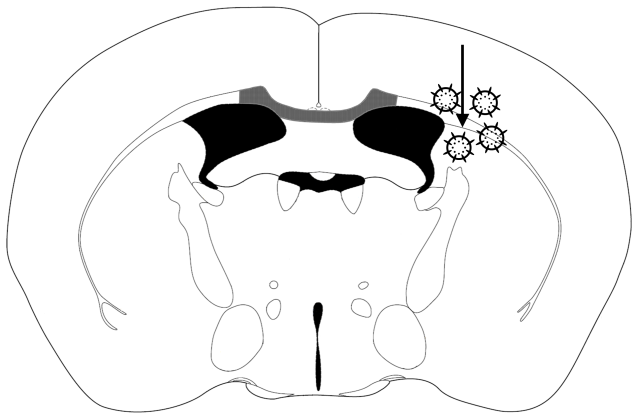
***

***Supplementary Fig. 1. Graphical representation of the region of interest (ROI) and the site of stereotactic injection.***

The mid region of the corpus callosum (shaded) was chosen for all analysis and is further referred as ROI in the text. For various quantifications in the ROI, at least three coronal sections of a mouse brain, 150 µm apart, obtained between the anterio-posterior coordinates of bregma -0.3 to -1.5 mm were analysed. Stereotactic injections in all experiments were performed using the coordinates anterio-posterior = -0.5 mm; lateral = -2.0 mm; dorso-ventral= -2.0 mm with respect to bregma (site of injection- arrow point). Graphic of brain: courtesy: mouse brain in stereotactic coordinates Paxinos and Franklin 2001.

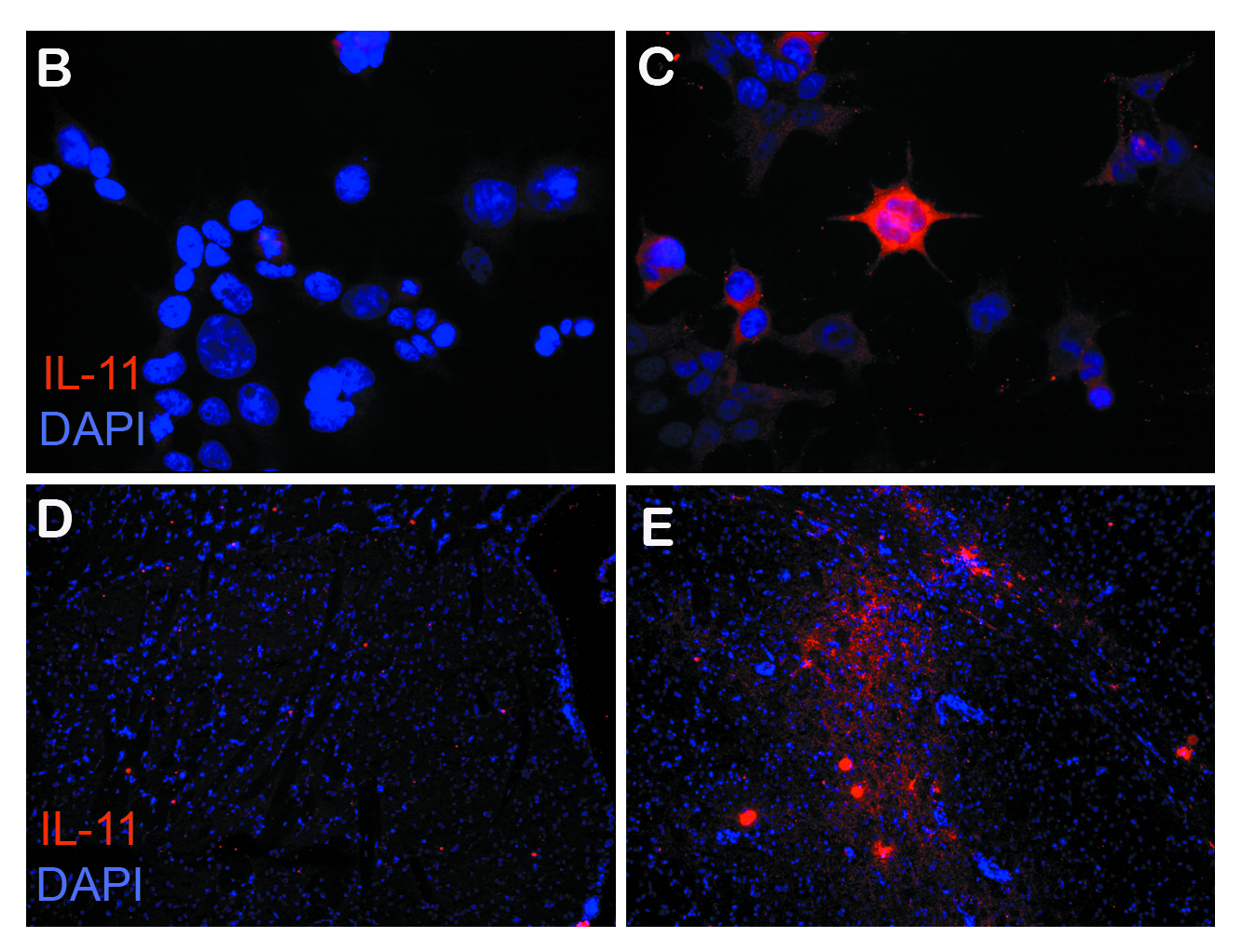


***Supplementary Fig. 2. In vitro and in vivo validation of transgene expression (IL-11) using LV***

For the *in vitro* validation of the transgene expression, HEK293T cells were transduced with IL-11-LV. Sham transduced cells were used as control. (A) ELISA of the supernatants collected from HEK293T cells transduced with IL-11-LV, 48 and 72 h post transduction, showed increased secretion of IL-11 by the transduced cells. Data is presented as mean ± SEM (n=3 biological wells for each group). Statistical significance was analyzed by One-way ANOVA followed by Bonferroni’s multiple comparison test. * *P*<0.05 with respect to SHAM control group. Immunocytochemical staining of Sham transduced cells (B) showed no expression of IL-11 while cells stained positively when transduced with IL-11-LV (C). For the *in vivo* validation of transgene expression, mice were stereotactically injected with 4µl of of IL-11-LV (at predefined coordinates). After 2 weeks of overexpression, brains of the mice were isolated and processed for immunostaining. While no IL-11 expression was observed in the contralateral hemisphere (D) a robust IL-11 expression was evident in the ipsilateral (E) hemisphere revealed high expression of IL-11 along the corpus callasum and striatum. No expression was observed in the contralateral hemisphere. Sham infected brain slices did not show a non-specific staining and served as control (data not shown).


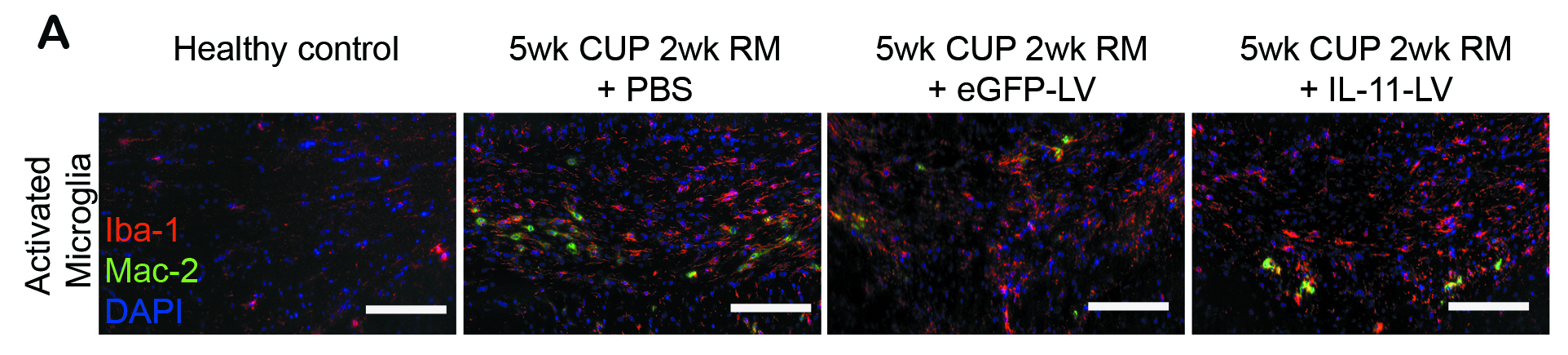

***Supplementary Fig. 3. Therapeutic overexpression of IL-11 downregulates the activation of microglia during remyelination.***

Quantitative analysis of the coronal brain sections reveals that therapeutic overexpression of IL-11 downregulates the activation of microglia in the corpus callosum, (A) Representative images of coronal brain sections, from all groups, depicting the density of activated microglia ( Iba-1^+^ /Mac2^+^ ) Quantitative analysis of double positive cells reveals that therapeutic overexpression of IL-11 decreases the density of activated microglia. Data is presented as mean ± SEM (n=5 animals for each cuprizone treated group; n=3 for healthy control group). Statistical significance was analyzed by one-way ANOVA followed by Bonferroni’s multiple comparison test. §§ *P*<0.01, §§§ *P*<0.001 with respect to healthy control group; * *P*<0.05, with respect to 5wk CUP 2wk RM + PBS; # *P*<0.05, with respect to 5wk CUP 2wk RM + eGFP-LV group. Quantification was performed in the corpus callosum of at least three coronal brain sections per mouse, chosen between anterio-posterior coordinates -0.3 to -1.5 mm (in reference with bregma).
